# Optimization of 4-Cyano-4’-pentylbiphenyl Liquid Crystal Dispersed with Photopolymer: Application Towards Smart Windows and Aerospace Technology

**DOI:** 10.3390/polym17162232

**Published:** 2025-08-16

**Authors:** Govind Pathak, Busayamas Phettong, Nattaporn Chattham

**Affiliations:** 1 Geo-Information and Space Technology Development Agency (GISTDA), Sriracha, Chonburi 20230, Thailand; govindpathak001@gmail.com; 2 Department of Physics, Faculty of Science, Kasetsart University, Bangkok 10900, Thailand; nattaporn.c@ku.ac.th

**Keywords:** liquid crystals, polymer, smart windows, transmittance, aerospace technology

## Abstract

The present reported work deals with the preparation of an energy-efficient smart window based on liquid crystal (LC) using a polymer-dispersed liquid crystal (PDLC) technique. The smart window was prepared using an LC–polymer composite by mixing photopolymer NOA-71 into nematic liquid crystal (NLC) 4-cyano-4’-pentylbiphenyl (5CB). The liquid crystal cell was prepared, the LC–polymer composite was filled inside the cell, and voltage was applied after the exposure of ultraviolet (UV) light. Textural analysis was carried out, and microscope images were taken out with the variation in voltage. Optical measurements were also performed for the smart window based on the PDLC system. Threshold voltage and saturation voltages were measured to carry out the operating voltage analysis. Transmittance was measured as a function of wavelength at different voltages. An absorbance study was also performed, varying the voltage and wavelength. The change in the power of the laser beam passing through the prepared smart window as a function of voltage was also investigated. The working of a prepared smart window using liquid crystal and a photopolymer composite is also demonstrated in opaque and transparent states in the absence and presence of voltage. The output of the present investigation into a PDLC-based smart window can be useful in the applications of adaptive or light shutter devices and in aerospace technology, as it shows the dual nature of opaque and transparent states in the absence and presence of electric field.

## 1. Introduction

Liquid crystals (LCs) are one of promising materials for the development of current scientific research. LCs are the intermediate phase of perfectly aligned crystals where molecules are aligned perfectly and randomly oriented liquids where the molecules show an isotropic nature [1,2,3,4,5]. LC materials opened a new research area for scientific researchers worldwide due to their wide range of applications. These materials are anisotropic in nature, which means that the properties of the liquid crystal depend on the direction of the molecules. The orientation of the LC molecules can be changed by applying a certain amount of electricity and temperature. This unique feature of anisotropy and small voltage requirements makes LCs very useful for the modern display and non-display devices [6,7,8,9]. Liquid crystal displays (LCDs) and flat panel displays (FPDs) are examples of liquid crystal applications that require high electro-optical and optical performance. The applications of liquid crystal in the making of flat panel displays have been reviewed by Hilsum et al. [10]. The development of modern FPDs and the physics and engineering behind it using liquid crystals is being widely discussed here. LC-based devices generally operate at a lower voltage when compared to the other materials. Kobayashi et al. have reported the different improvements in the physical parameters of liquid crystal features of LCDs [11]. The applications of liquid crystals beyond displays such as sensors, lenses and supercapacitors have been reported by Devadiga et al. [12]. The smart window is also one of the crucial non-display application examples where transparency can be tuned by applying a definite amount of voltage and temperature [13]. There are generally two types of liquid crystals: thermotropic and lyotropic [14,15,16]. Thermotropic liquid crystals are temperature dependent, whereas lyotropic liquid crystals are concentration dependent. Thermotropic LCs are very useful due to their thermal and optical features. When the temperature of the thermotropic liquid crystal rises, then it shows further different phases such as nematic [17,18] and smectic [19] phases, and then it reaches an isotropic point where the LCs behave as ordinary liquids when the temperature further increases. NLCs are easily aligned in the presence of an electric or magnetic field. Nematic LCs are widely studied due to their applicability toward display and non-display devices. Alipanah et al. have reported on the temperature-dependent optical properties of NLCs [20]. The measurement of the refractive index and order parameter of NLCs has been investigated. The anisotropic wave propagation in nematic liquid crystals was also studied by Biscari et al. [21]. The anisotropy of sound velocity and its frequency dependence is explained by gradient continuum theory in this investigation.

The orientational and optical properties of the nematic phase of liquid crystals can be tuned by mixing suitable additives into it. The dispersion of different dopants such as nanoparticles (NPs) [22,23,24], dye [25] and polymers [26] changes the electro-optical, dielectric and optical parameters of liquid crystals. This is the easy and cost-effective way to tune the physical properties of LCs without affecting their viscosity and stability. Among these guest–host systems, the mixing of polymers into liquid crystal proves to be very valuable in the liquid crystal-based smart devices, as it generates an opaque and transparent state which works as an adaptive or smart window [27,28,29]. The smart window blocks the light and also allows passing through it in the absence and presence of an electric field. Therefore, research work on the smart windows using polymer-dispersed liquid crystal is nowadays crucial for diverse scientific advancement. Hicks et al. have reported on mixing the polymers into nematic liquid crystals to study electric polarization under externally applied electric fields [30]. Shanks et al. have investigated the thermal and optical characterization of PDLC systems [31]. Cuevas et al. have reported on the frequency-driven self-organized helical superstructure and showed that transparent and scattering states can be observed in nanoparticle-embedded liquid–crystal systems [32]. Investigations of the mixing of different dopants into a PDLC system using three-component systems to improve the dielectric and thermal parameters of LC-based devices were recently reported [33,34]. The mixing of photopolymers into liquid crystal is also an important technique for the fabrication of smart windows because a photopolymer polymerizes when it is exposed under UV light. The present reported work deals with the dispersion of photopolymerizable polymers into nematic liquid crystals to make them work as energy-efficient smart windows based on a polymer-dispersed liquid crystal system. Microscope textural and optical analyses like transmittance and absorbance have been performed to analyze the smart window.

## 2. Materials and Methods

### 2.1. Materials

#### 2.1.1. Liquid Crystalline Material

The selection of liquid crystal plays an important role in the preparation of the PDLC-based smart window because the refractive index and anisotropic behavior change the characteristic of the device. The liquid crystalline material used in the present investigation is 4-cyano-4’-pentylbiphenyl (5CB), which is a nematic liquid crystal, and it was purchased from Sigma Aldrich. This material shows the nematic liquid crystal phase at 22.5 °C and goes to the isotropic state at 35 °C. The temperature phase sequence of the used 5CB NLC is as follows:

Cr 22.5 °C N 35 °C I

**Figure 1:**
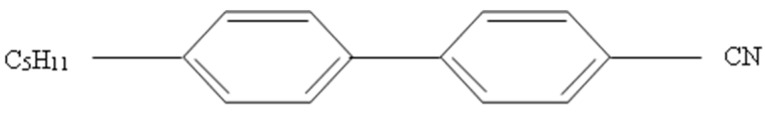
Chemical structure of nematic liquid crystal 4-cyano-4’-pentylbiphenyl (5CB).

Here, ‘Cr’, ‘N’ and ‘I’ represent the crystal, nematic and isotropic phase of the liquid crystal. The chemical formula for the 5CB nematic liquid crystal is C_18_H_19_N and the chemical structure is shown in Figure 1. The used 5CB liquid crystal here is a positive dielectric anisotropy material. The purity of the used liquid crystal is ≥98%. The refractive index of this LC at room temperature is 1.58 for the ordinary refractive index (n_o_) and 1.77 for the extraordinary refractive index (n_e_), and the resulting birefringence is 0.20 [35].

In the molecular arrangements of the 5CB liquid crystal, molecules are perfectly arranged and oriented in a certain direction and position in the crystalline state. However, when the temperature is increased, the orientation of the LC molecules changes with no positional order, and it reaches the nematic phase of the liquid crystalline state. The nematic phase carries only an orientational order and not the positional order. When the temperature is further raised to its isotropic point, then the molecule acquires a random orientational and positional order and goes to the isotropic phase where it behaves as a general liquid.

#### 2.1.2. Polymer Used

The polymer used in the present reported work was the commercial photopolymer Norland Optical Adhesive (NOA-71), and it was purchased from Norland Products, Inc. This photopolymer NOA71 is a urethane-based formulation containing mercapto-easter. It is a clear liquid adhesive which cures under UV light and provided maximum adhesion and moisture resistance when used in glass. Its viscosity at room temperature is 200 cps, and the dielectric constant at 1 MHz is 4. This photopolymerizable polymer was mixed with liquid crystal to fabricate the PDLC-based smart window. This optically clear liquid adhesive photopolymer readily polymerizes when exposed to UV light for a few minutes. The refractive index of the cured polymer NOA71 is around 1.56. The refractive index of the polymer and liquid crystal are different in the mixture and match when voltage is applied within the filled LC cell.

### 2.2. Mixing of Liquid Crystal and Polymer

To make the PDLC mixture for the fabrication of the smart window, the nematic liquid crystal (5CB) was mixed with the photopolymer NOA71 in a 60:40 ratio at room temperature. The concentration of the liquid crystal and polymer in the PDLC mixture is crucial, as it affects the orientational order and optical properties of the material. Usually, a high concentration of photopolymer compared to the liquid crystal content in the PDLC mixture increases the driving voltage required to switch the PDLC device, which causes the high energy consumption. However, a smaller concentration of polymer than that of LC can reduce the driving voltage, but it does not create a good contrast of opaque and transparent condition, as it reduces the opacity of the device even in the absence of voltage. Therefore, a medium and optimum concentration of polymer, slightly lower than the LC content, can be used to obtain a good opaque and transparent state in the OFF and ON condition as well as achieve the low driving voltage. That is the reason for the concentration of photopolymer used in the present reported work, 40%, which was mixed with 60% liquid crystal to prepare the PDLC mixture. This mixture was prepared by mixing LC and polymer for 2 h at room temperature.

### 2.3. Fabrication of Liquid Crystal Cell

The liquid crystal cell was prepared using glass plates to fabricate the PDLC-based smart window. Two indium tin oxide (ITO) glass plates were used to prepare the liquid crystal cell to use it as a smart window. The resistance of the ITO glass plate was ~40 Ω. The glass plate was highly transparent and uniformly flat. ITO plates were first cleaned by hot soap solution (60 °C) to remove the organic impurities on the substrates, and then it was finally washed by acetone. Then, the plates were put inside the oven for 1 h at 80 °C. Afterwards, the glass plates were removed from the oven and cooled down to room temperature. Thereafter, the conducting sides of the plates were checked using a digital multimeter, since the glass plates are conducting on one side and non-conducting on the other. Then, the assembling process was started where two ITO plates were assembled using optical glue mixed with the 10 μm mylar spacer. Both plates were put over each other and exposed to UV light for curing for 10 min. There was no alignment on the plates, and therefore no rubbing was required. After UV curing, the glass plates stuck to each other and then were kept at room temperature for 24 h to make the curing more effective.

### 2.4. Filling of LC–Polymer Mixture in LC Cell and UV Exposure: Smart Window

**Figure 2:**
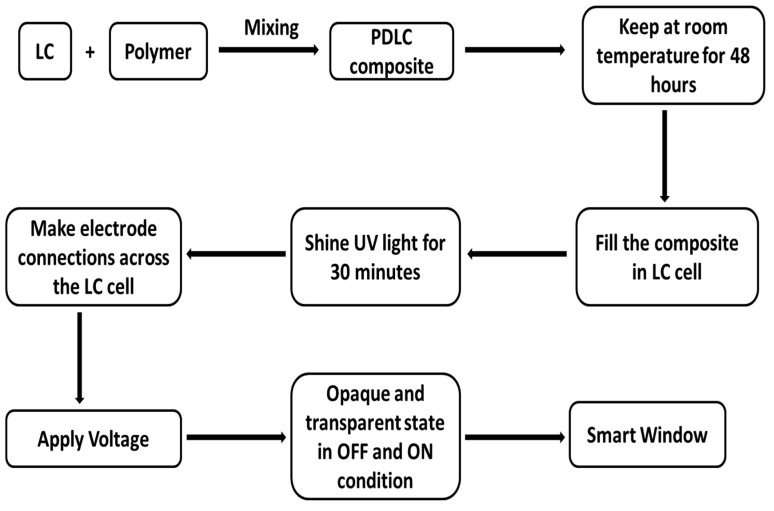
Schematic diagram for the preparation of PDLC-based smart window.

The liquid crystal–polymer mixture in a 60:40 ratio was filled into the liquid crystal cell using capillary action. The cell looks transparent at this stage, and after filling the PDLC mixture inside the cell, ultraviolet light of 365 nm and 6 W power was shined onto the cell for 15 min. This UV exposure polymerizes the photopolymer, and droplets of LC molecules are created within the matrix. The cell becomes opaque at this stage after the UV exposure. Then, the electrode connection was made onto the liquid crystal cell. The copper tape and wire was used for the electrode connection. The electrical connection can be made through electrodes. Finally, 40 V voltage was applied, and the cell became transparent. The cell turned to an opaque state when the voltage was removed. Therefore, this prepared cell filled with liquid crystal and polymer behaves as a smart electrically controlled smart window, where its transparency can be tuned by applying a certain amount of voltage. A schematic diagram of the preparation of the PDLC-based smart window is demonstrated in Figure 2. The step-wise process of making the composite, filling the composite in the LC cell, the shining of UV light and application of voltage across the cell to work as a smart window are depicted.

### 2.5. Instruments Used

The textures of the liquid crystal and polymer composite were taken using a Zeiss Microscope (Axio, München, Germany). A CS2020 UV LED curing system (Thorlabs, Newton, NJ, USA) with 365 nm wavelength and 5 W power was used to shine the UV light to polymerize the liquid crystal–polymer composite. Transmittance and absorbance measurements were performed using a Shimadzu UV-1800 UV-Vis spectrophotometer (Shimadzu, Kyoto, Japan). A HT3005PB DC power supply was used to apply the voltage across the cell (Hantek, Qingdao, China). A Maestro power and energy meter was used to measure the power of the laser beam having a wavelength of 532 nm (Gentec-E, Quebec city, QC, Canada).

## 3. Results and Discussion

### 3.1. Textural Study

**Figure 3:**
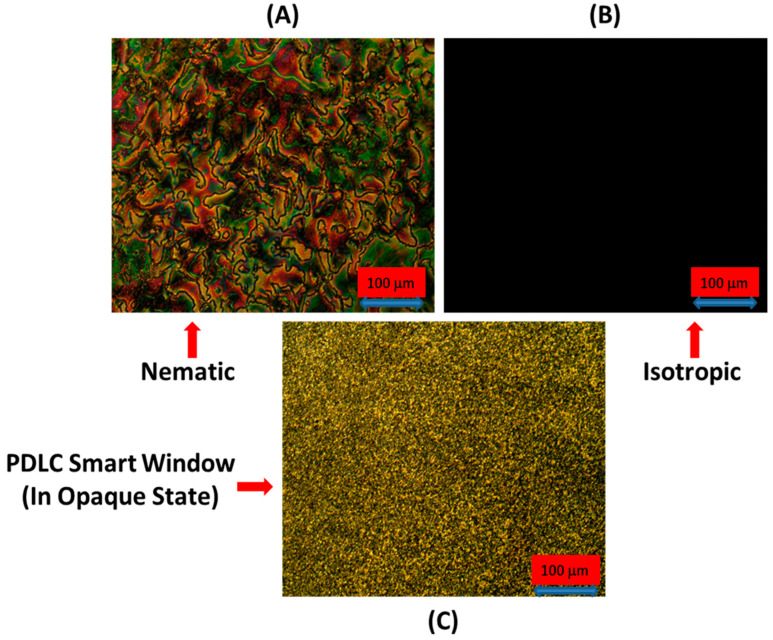
(**A**) Microscope Schlieren texture of untreated pure 5CB liquid crystal (nematic phase) at room temperature, (**B**) microscope texture of 5CB liquid crystal at isotropic point (35 °C) on unaligned glass plates covered with glass cover slip under crossed polarizer and analyzer condition, (**C**) microscope texture of PDLC smart window at room temperature in opaque state in absence of voltage.

Analysis of the microscope textures of the liquid crystalline material provide insightful information about the phase, alignment and orientation of the LC molecules. Therefore, textural analysis was performed in this study, as shown in Figure 3, for the pure liquid crystal and PDLC-based smart window. The textures shown were not additionally forced by surface-stabilizing actions of the nematic. Figure 3A depicts the microscopic schlieren texture of the 5CB liquid crystal at room temperature, which represents the nematic phase of the liquid crystals. The Schlieren texture confirms that the 5CB liquid crystal shows a nematic phase at room temperature. The textures in the A and B images were taken by putting the pure liquid crystal between the glass slide and cover slip under a crossed polarizer and analyzer condition. Figure 3B shows the microscope texture of the LC by raising the temperature up to its isotropic point (35 °C). The dark black texture shows that the molecules have a random positional and orientational order and go into the isotropic state. The microscope texture of the filled LC cell (PDLC-based smart window) in the opaque state is shown in Figure 3C. The LC cell for POM textures was prepared using ITO glass plates. The plates were cleaned, and optical glue was put on the side area of the conducting region of the plates. Both plates were assembled by putting a spacer between them to make a cell, and a liquid crystal–polymer composite was sandwiched between the plates of the cell. This texture was recorded under the crossed polarizer and analyzer condition at room temperature in the absence of voltage. A high-density droplet image with bright color was observed due to the high content of liquid crystal in the polymer matrix. Voltage wise, a change in the texture of the prepared smart window was also carried out.

**Figure 4:**
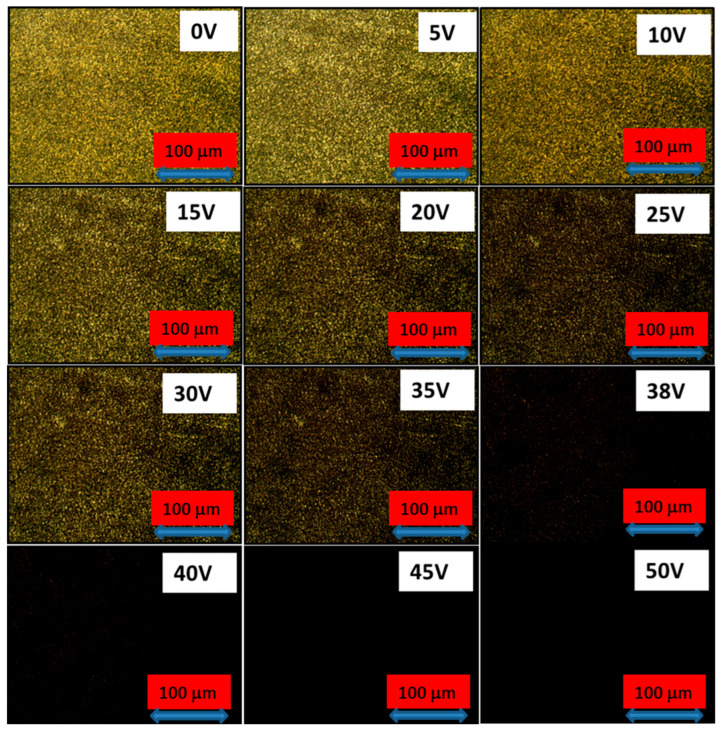
Microscope textures of PDLC system-based smart window at different voltages in crossed polarizer and analyzer condition.

Figure 4 depicts the microscope textures of the prepared PDLC smart windows with the variation in voltage at room temperature under crossed polarizer and analyzer conditions. It is clearly seen in the figure that at 0 V, the PDLC smart window shows high density droplet textures with visible brightness due to the high content of liquid crystal when compared to the polymer content. The LC forms droplets when exposed to the ultraviolet light in the presence of photopolymer. When the voltage is increased to 5 and 10 V, then the brightness and droplet density starts decreasing, which means that the opacity of the smart window is reducing and transparency is increasing by increasing the voltage. Moreover, when the voltage is increased further up to 30 V, the brightness reduces with the droplet density. At 38 V voltage, we can clearly see that most of the space filled with darkness, because the smart window becomes almost transparent. A saturation point is observed at 40 V where a complete black texture can be seen, because at this voltage, the prepared PDLC-based smart window completely turns into a transparent state. This transparent and opaque nature was confirmed by the optical transmittance measurement, which will be discussed later.

### 3.2. Optical Study

**Figure 5:**
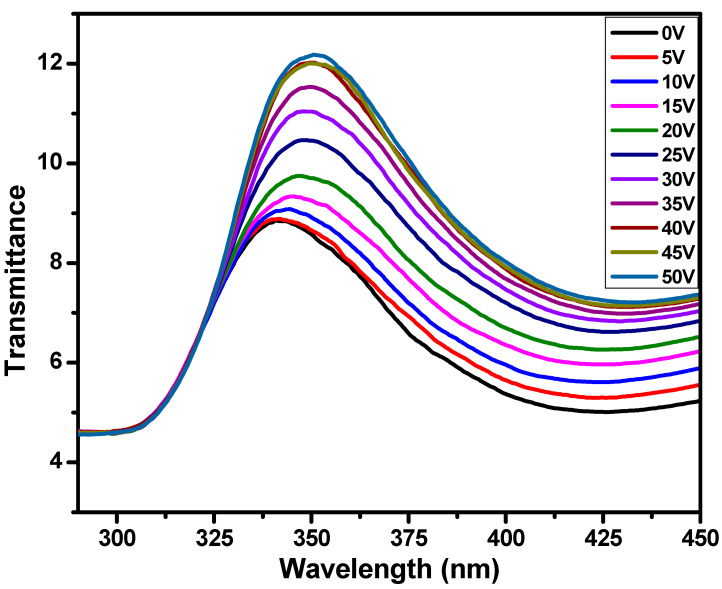
Change in transmittance as a function of wavelength with the variation in voltage for PDLC system-based smart window.

The optical parameters of the polymer-dispersed liquid crystal-based smart window were also measured. The transmittance of the prepared smart window was measured by voltage and wavelength variation. A change in transmittance as a function of wavelength in arbitrary unit and voltage is shown in Figure 5. It can be seen from this figure that the transmittance increases as the voltage increases. The black curve represents the transmittance at 0 V at an opaque state of the smart window. When the voltage is increased, then the transmittance peaks are increasing, and after a certain amount of voltage (~40 V), it becomes almost saturated. This means that near 40 V voltage, the smart window becomes transparent, and all light passes through the smart windows. When no voltage is applied, that is when the smart window is in an opaque state; then, there is a mismatch between the refractive index of liquid crystal and polymer. As a result, light scattering occurs, and it shows an opaque state. However, when the voltage is increased, the mismatch between the LC molecules and polymer started reducing, and hence its opacity reduces and transparency increases. When the voltage reaches almost 40 V, then there is no mismatch of refractive indices between the 5CB LC molecules and NOA71, and at this voltage, the orientation is saturate. The refractive index of both the LC molecules and polymer matches completely, and the smart window show no scattering of light, but it becomes transparent. The linear shifting of wavelength toward a higher region is also observed for the increased voltages. This higher side of wavelength shifting might be due to the coupling of incident photon and liquid crystal molecules and change in the orientation of LC droplets along the electric field when the voltage increases. This dual state smart window device changes its state from opaque to transparent by applying the voltages, which is crucial for many adaptive light applications like offices and aerospace, where it can replace plane windows.

**Figure 6:**
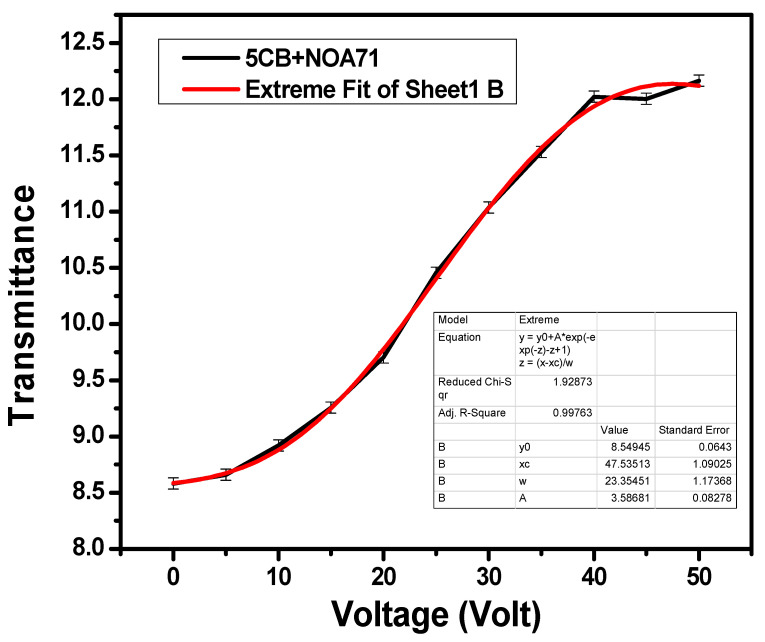
Change in transmittance as a function of voltage at 350 nm wavelength for PDLC system based smart window. Typical error bars are shown. Red curve represents the fitted data of transmittance.

Figure 6 shows the variation in transmittance as a function of voltage at 350 nm wavelength. It can be clearly seen from this figure that the transmittance values starts increasing after 5 V and reach saturation at 40 V. These data were also fitted using graphical representation software, and the fitted curve can be seen in the red line. At 0 V, the prepared smart window based on the polymer-dispersed liquid crystal method shows an opaque state and transformed into a transparent state near 40 V. At higher voltages, the refractive indices of the LC and polymer match, light scattering reduces, and light passes throughout the PDLC; hence, it becomes transparent in nature.

**Figure 7:**
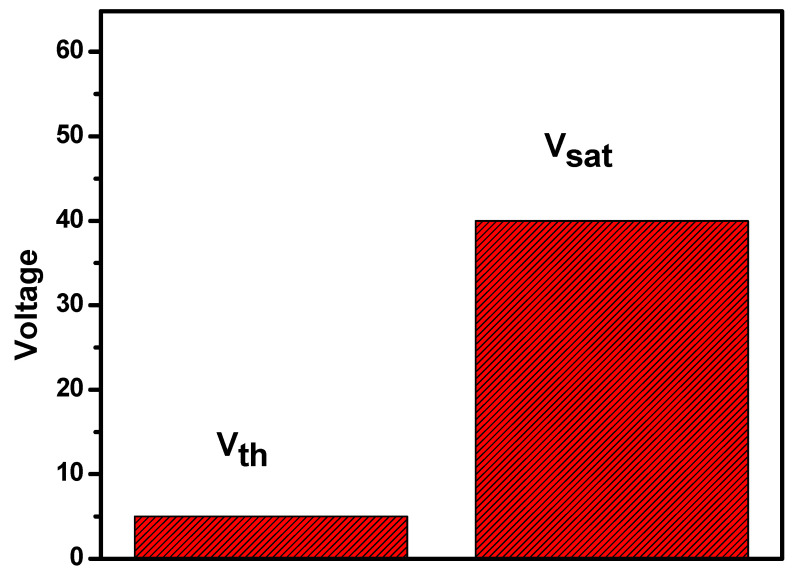
Comparative chart of threshold voltage (Vth) and saturation voltage (V_sat_) for the PDLC smart window.

The change in the transparent state from the opaque state starts from the threshold voltage (Vth) of the PDLC-based smart window, and it completely becomes transparent at its saturation voltage (V_sat_). At the threshold voltage, the orientation of LC molecules starts changing, and the orientation of the LC matrix completes at the saturation voltage. The refractive index of the PDLC system completely matches at this point, and hence the transparent state occurs. The observed threshold voltage for the fabricated smart window was found to be near 5 V, and the saturation voltage occurred near 40 V. The comparative demonstration of threshold and saturation voltage is shown in Figure 7.

**Figure 8:**
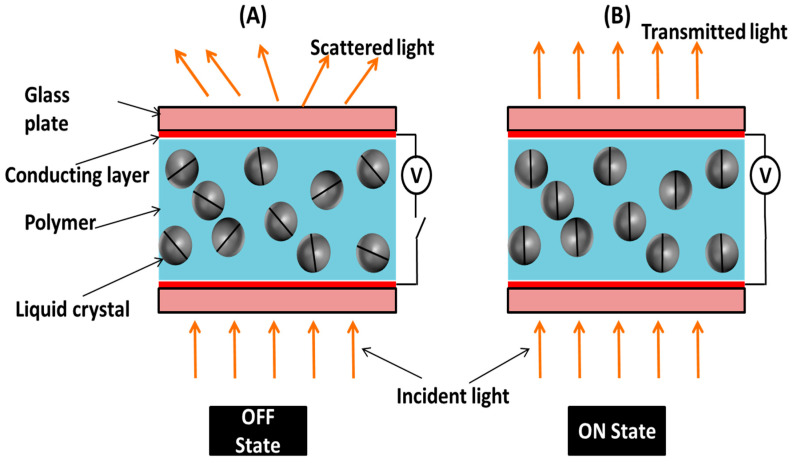
Schematic representation model of working of prepared smart window based on PDLC system, (**A**) PDLC-based smart window in OFF state, (**B**) PDLC-based smart window in ON state.

In the PDLC system, there is a mismatch between the refractive index of liquid crystal molecules and the polymer in the OFF condition, so light scatters when falls onto it, and it shows an opaque state. When voltage is applied, then the refractive index of the liquid crystal molecules and polymer matches, light passes through it, and a transparent state is observed. The schematic representation model of the prepared smart window in the ON and OFF states is demonstrated in Figure 8. The prepared PDLC smart window operates at around 40 V, where the transparency becomes saturated. It switches its state from opaque to transparent condition completely when 40 V voltage is applied across the electrodes of the device. Figure 8A shows the interaction of light in the PDLC-based smart window in the OFF condition when there is no voltage applied, and Figure 8B represents the light interaction with the PDLC system in the ON condition when 40 V voltage is applied. The PDLC cell is made up of two conducting glass plates, and the liquid crystal–polymer composite is sandwiched between the substrates. It is seen from Figure 8A that the LC droplets have a random orientation in the OFF state and therefore cause the light inside the PDLC system to disperse. This is the opaque state of the PDLC smart window. Figure 8B demonstrates that when the voltage is increased up to its saturation point (~40 V), the orientation of LC droplets changes in the direction of the electric field in the ON state. At this state, the refractive indices of the polymer and LC coincide, it becomes completely transparent, and light passes through the PDLC system. Therefore, the observance of two states in the OFF and ON conditions makes this PDLC system operate as an electrically controlled smart window.

The prepared smart window is electrically switched between transparent and scattering states. The PDLC smart window is opaque in the absence of an electric field due to the difference in refractive index between the LC droplets and the polymer matrix. The used LC material has the positive dielectric anisotropy, so the molecules rearrange along the electric filed direction when sufficient voltage is applied, and the PDLC smart window becomes transparent. This transparent state occurs because of matching between the ordinary refractive index of the liquid crystal (n_o_) and the refractive index of the polymer (n_p_) matrix [36]. When the voltage is removed, then the PDLC smart window returns to its opaque state due to the strong anchoring effects of the molecules of liquid crystal from the polymer matrix.

**Figure 9:**
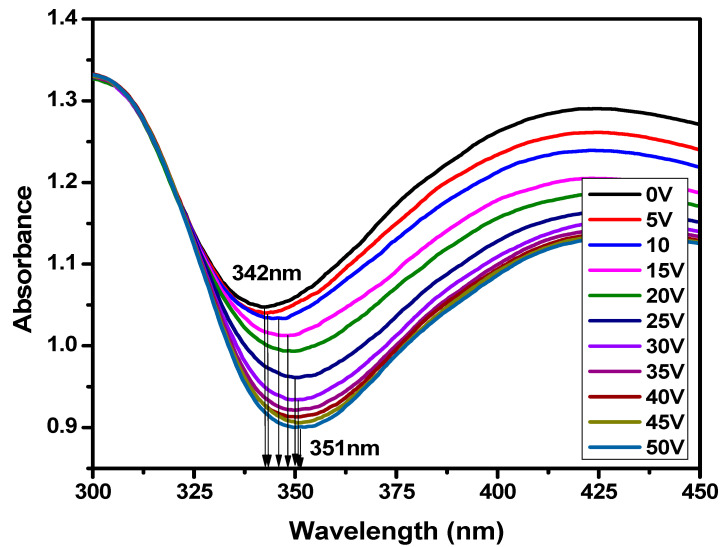
Change in absorbance as a function of wavelength with the variation in voltage for PDLC system-based smart window.

Figure 9 shows the change in UV absorbance as a function of wavelength with the variation in voltage of the PDLC-based smart window. The absorbance peak for the opaque state was found to be at 342 nm. The π–π excitation of the π-electron system of the polymer and liquid crystal composite is the primary reason for the absorption peak in the UV region. It is shown in the figure that absorbance decreases as the voltage increases. This result confirms the transmittance results, as discussed earlier. The absorbance starts decreasing when the voltage is increased from 0 V, and it becomes saturated near 40 V where the smart window completely changes its state from opaque to transparent. In the transparent state, there is a minimum absorbance of light, and in the opaque state, there is a maximum absorbance of light observed. This confirms the two states of the fabricated smart window, which can change its state by applying voltage. The absorbance peak at 0 V was found to be near the 342 nm wavelength, and the absorbance peaks starts shifting at the higher side of the wavelength when the voltage is increased. At the saturation voltage (~40 V) and above the saturation voltage, the absorbance peaks were near 351 nm. This result shows that the absorbance peaks shifted toward a higher wavelength from 342 nm in the opaque state to 351 nm in the transparent state with the application of voltage.

**Figure 10:**
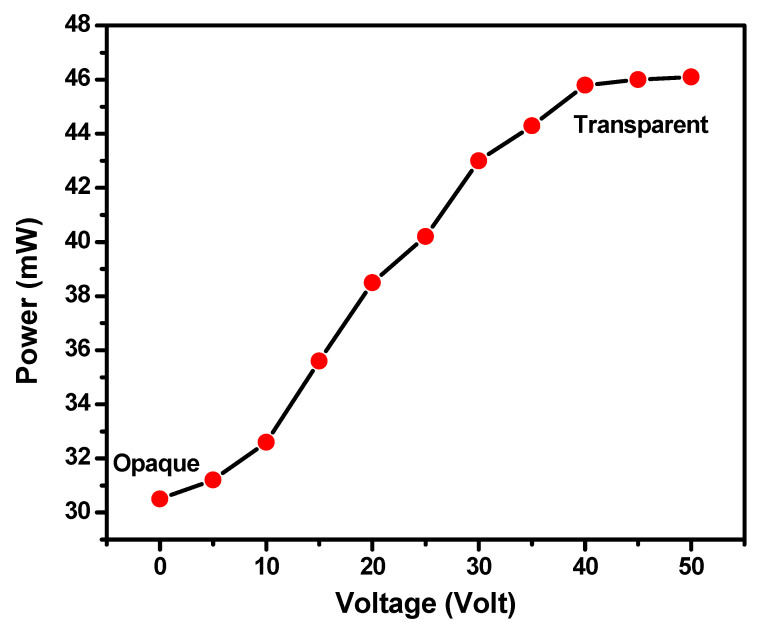
Change in power of laser beam as a function of voltage for PDLC smart window.

The power of the laser beam passing through the PDLC system-based smart window in the opaque and transparent state was also investigated. Figure 10 depicts the change in the power of the laser beam with the variation in voltage at room temperature. The power of light is found to be minimum in the opaque state (at 0 V) and starts increasing when the voltage is increased, and then it becomes saturated when the smart window comes in the transparent state near 40 V. This trend in the power change indicates that the opaque state of the smart window is blocking some of the laser light and allows the light to completely pass through it in the transparent condition. Therefore, this result confirms that the prepared smart windows can be used as a light shutter, which may also be applicable in aerospace technology.

**Figure 11:**
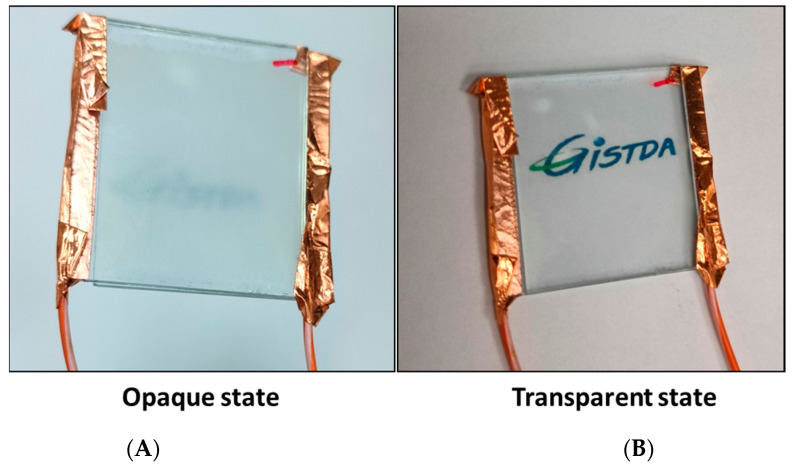
Demonstration of PDLC-based smart window in opaque state and transparent state. (**A**) In opaque state at 0 V, letters the “GISTDA” cannot be seen clearly. (**B**) In transparent state at 40 V, the letters “GISTDA” can be clearly seen.

The final prepared PDLC smart window in the opaque state and ON transparent state is shown in Figure 11. The opaque and transparent state was observed in the absence and presence of voltage. The prepared PDLC works as a smart window where its transparency can be tuned by applying a certain amount of voltage. It can be seen from this figure that when it is in the OFF condition without an electric field, then it is in the opaque state, and when the electricity is applied, then it becomes transparent. In the OFF condition, we cannot see the letters “GISTDA” clearly by eye because this smart window blocks the light from passing through it. However, when 40 V voltage is applied, then it becomes transparent, light passes through it, and the letters “GISTDA” can be clearly seen. Therefore, it can be said that the prepared PDLC liquid crystal cell works as an electrically controlled smart window. This is an energy-efficient smart window and can be operated at lower voltage when compared to the general PDLC adaptive glasses, which operate at much higher voltages.

The output of the present reported work can be crucial for energy-efficient electrically controlled smart windows. The reported liquid crystal and polymer composite is chemically stable and easy to prepare, as it does not require a long time to become polymerized in the presence of UV light. The fabricated smart window device is also electrically stable for high and low voltages for long periods of time. The transparency and opacity along with the microscope textures of the device were checked in the range of one to four months and it showed similar results for the whole period of time, which means that degradation of the mixture had not occurred. So, its longevity and ability to bear the continuous voltage operation did not affect the device operation, which indicates its good stability. The observed change from the opaque state to transparent state with a lower amount of voltage than that of normal PDLC adaptive glasses makes the obtained results beneficial for the smart window research area. Significant increments in the transmittance and decrements in the absorbance for the reported smart window in the presence of electric field is desired for LC-based smart devices.

## 4. Conclusions

Liquid crystal materials are anisotropic in nature, and the properties of the LCs depend upon the direction of molecules. In the present reported work, an LC-based smart window was prepared, and its optical properties and textural measurements were investigated. The prepared electrically controlled smart window shows two states: opaque and transparent in the absence and presence of voltage. A microscope textural analysis of the PDLC-based smart window with the variation in voltage at room temperature revealed a bright droplet texture of LCs in the polymer matrix was observed at a zero voltage condition. When voltage was applied, then the droplet density reduced, and it showed dark textures in a transparent state near 40 V. Optical measurements were carried out, which confirmed the two state behaviors of the fabricated smart window. A transmittance study for the PDLC-based smart window as a function of wavelength at different voltages showed an increment in the transmittance when the voltage was increased. The threshold voltage was found to be near 5 V and the saturation voltage was found to be near 40 V. An absorbance study as a function of wavelength revealed that absorbance starts decreasing as the voltage increases. The absorbance of light decreased when the LC molecules oriented themselves along the direction of the electric field. The power of the laser beam passing through the smart window was measured and found to be increased for the transparent state when compared to the opaque state. The final prepared smart window also demonstrated opaque and transparent states, and light-adaptive behavior can be clearly seen. The reported liquid crystal-based electrically controlled smart window is an energy-efficient device which can operate at low voltage. It can block the light as well as allow the light to pass through according to requirements, which can be used as a light shutter for different applications. This may also be useful in aerospace technology, where it can be used in the windows of aeroplanes and payloads due to its lower weight and ability to operate at a lower voltage.

## Data Availability

The original contributions presented in this study are included in the article. Further inquiries can be directed to the corresponding author.
